# Clinicopathological features of adult right-sided cardiac masses: Analysis of 19 cases

**DOI:** 10.1016/j.amsu.2022.103613

**Published:** 2022-04-10

**Authors:** I. Lahmidi, C. Darar Assoweh, I. Haddiya, Y. Bentata, N. El ouafi, N. Ismaili

**Affiliations:** a Department of Cardiology, Laboratory of Epidemiology, Clinical Research and Public Health, Medical School, University Mohammed the First, Oujda, Morocco; b Department of Nephrology, Laboratory of Epidemiology, Clinical Research and Public Health, Medical School, University Mohammed the First, Oujda, Morocco

**Keywords:** Adults, Right sided cardiac masses, Echocardiography, Case series

## Abstract

**Background:**

Right sided cardiac masses are rare. The purpose of this study is to review the clinical experience and pathological characteristics of right-sided cardiac masses and to provide a prognostic analysis in our hospital.

**Methods:**

We retrospectively reviewed 19 consecutive cases of right heart masses diagnosed in our institution from 2016 to February 2020. All available clinicopathological features, imaging characteristics and disease outcomes were summarized and presented.

**Results:**

The subjects included 9 men and 10 women with a mean age of 48.5 years. The most frequent complaint was dyspnea. The most common site was the right atrium (42.1%) followed by the tricuspid valve (36.8%). Clinical diagnosis revealed vegetations in 8 patients (42.1%), thrombi in 7 patients (36.8%), myxoma in 1 patient, hydatid cyst in 1 patient and metastatic (secondary) masse was seen in 2 cases. In the 19 patients, 3 patients underwent surgery, 15 patients were managed with medical treatment, therapeutic abstention was indicated in one patient. 14 patients were all alive at the end of the follow-up period. In contrast, 5 patients were dead (26.3%).

**Conclusion:**

In our series, the majority of right cardiac masses were benign, outnumbering the malignant ones, as described in the literature. The mortality rate was relatively high about 26.3%.

## Highlights

• Right sided cardiac masses represent a rare condition. They include a large spectre of lesions with different clinical, etiological and radiological features.
• In this article, we provide a retrospective study of nineteen patients with right side cardiac masses initially diagnosed with TTE. As reported in literature, the most common causes were non-neoplastic lesions such as vegetations (42,1%) and thrombi (36,8%).
• Due to the absence of a uniform clinical presentation, having a high index of suspicion, non-invasive imaging and a reasonable approach are important means for an appropriate diagnosis.

## 1 Introduction

Right-sided cardiac masses are infrequent with significant heterogeneity in pathology and clinical presentation [1]. They encompass a broad set of lesions that include both neoplastic and non-neoplastic conditions. Metastatic tumors usually arise from lung, breast, melanoma and hematologic malignancies such as lymphoma [2]. Right-sided cardiac masses, such as thrombi and vegetations, can often mimic normal cardiac structures. The evaluation of cardiac masses may therefore be a diagnostic challenge, therefore a step-by-step approach may serve well practically. Timely and accurate diagnoses of cardiac masses are important with respect to providing early effective treatment and facilitating good outcomes.

The following discussion details the findings of nineteen cases of right intracardiac masses, diagnosed initially by echocardiography, and reviews the relevant literature.

## 2 Materials and methods

This is a descriptive and retrospective, single center study of all consecutive patients admitted to our cardiac care unit from 2016 to February 2019. We identified 19 patients with right-sided cardiac masses diagnosed by transthoracic echocardiography. All patients clinical data and imaging features were retrospectively reviewed.

Transthoracic Echocardiography (TTE) was performed on all of the patients as the initial diagnostic procedure. Four patients also underwent *trans*-esophageal echocardiography (TEE). Cardiac MRI was performed in one patient. Vascular Doppler studies, D-dimer assay (by enzyme linked immunosorbant assay [ELISA]) and three blood cultures were carried out according to the clinical context. Computed tomographic pulmonary angiography was done in 18 patients where high pulmonary artery (PA) pressures or dilation of right ventricle (RV) or excessive mobility of the mass pointed to the possibility of associated pulmonary thromboembolism.

This case series has been reported in line with the PROCESS criteria [3].

## 3 Results

The salient clinical features of these patients are described in Table 1. As respects characteristics of the patients, female patients predominated with 52.6% of the total number of right cardiac masses (9 males versus 10 females with a male/female ratio of 0.9). The mean age of these patients was 48.5 years (age range: 18 years–91 years; median age: 45 years). Our study showed that the prevalence rate of right cardiac masses was quite different among different age groups, with the highest number of cases in the age group ≥60 years. Clinically the most common initial presenting symptom was dyspnea, reported by 57.9% of the patients. Other common symptoms include chest pain, palpitations fatigue and weight loss. Eight patients had fever (42.1%).

**Table 1:** Clinical and pathologic characteristics of right-sided cardiac masses.

Case	Age (years)/Sex	Presentation	Location	Pathologic diagnosis	Size (cm)	treatment
Case 1	74/Female	Chest tightness and pain, fever TV	Vegetation		_	Antibiotics
Case 2	45/Male	Dyspnea	RA	Thrombus	_	Anticoagulants
Case	58/Male	Dyspnea	RV	Thrombus	_	Anticoagulants
Case 4	22/Female	Dyspnea	RA	Thrombus	2.0 × 1.6	surgery
Case 5	66/Male	Dyspnea, fatigue, weight loss	RV	Metastasis	3.6 × 2.6	–
Case 6	18/Female	Dyspnea, fever	TV	Vegetation	1.3 × 0.8	Antibiotics
Case 7	72/Female	Dyspnea	RA	Thrombus	_	Anticoagulants
Case 8	88/Female	Dyspnea	RA	Thrombus	2.9 × 1.7	Anticoagulants
Case 9	21/Female	Fever	RA port catheter	Vegetation	1.8 × 0.4	Antibiotics + ablation
Case 10	47/Male	Fever, fatigue	TV	Vegetation	3.3 × 3.2	surgery + Antibiotics
Case 11	23/Male	Dyspnea, fever	TV	Vegetation	2.2 × 1.0	Antibiotics
Case 12	44/Female	Dyspnea	RA	Myxoma	2.9 × 2.1	–
Case 13	60/Female	Fever	RA	Vegetation	2.1 × 0.7	Antibiotics
Case 14	28/Female	Fever, fatigue	TV	Vegetation	1.5 × 0.7	Antibiotics
Case 15	91/Male	Palpitations	RA	Thrombus	4.2 × 2.2	Anticoagulants
Case 16	62/Male	Dyspnea	RA	Thrombus	_	intravenous thrombolysis
Case 17	32/Male	Dyspnea	RV	Metastasis	8.0 × 7.9	surgery
Case 18	34/Female	Fever	TV	Vegetation	2.4 × 1.5	Antibiotics
Case 19	38/Male	Asymptomatic	RV	Hydatid cyst	5.0 × 2.9	Therapeutic abstention

Electrocardiographic abnormalities were observed in 11 patients: four cases with atrial fibrillation, five with repolarization abnormalities and two with left ventricular hypertrophy. The imaging modalities utilized for evaluation of right-sided cardiac masses included TTE (100%), TEE (21.1%), and CMR (5.3%) (Table 2). The majority of right-sided cardiac masses were located in the right atrium (8 cases, 42.1%), seven arose from tricuspid valve (36.8%), and four from the right ventricle (21.1%). The size ranged from 8 × 7.9 to 1.3 × 0.8 cm, with most lesions <3 cm.

**Table 2:** Cardiac imaging of 19 patients diagnosed with Right-sided cardiac masses.

Cardiac imaging	Values
Identification of masses by imaging modality	
TTE	19/19 (100%)
TEE	4/19 (21.1%)
CMR	1/19 (5.3%)

Key: CMR, Cardiac magnetic resonance; TEE, Transesophageal echocardiogram; TTE, Transthoracic echocardiogram.

**Fig. 1:**
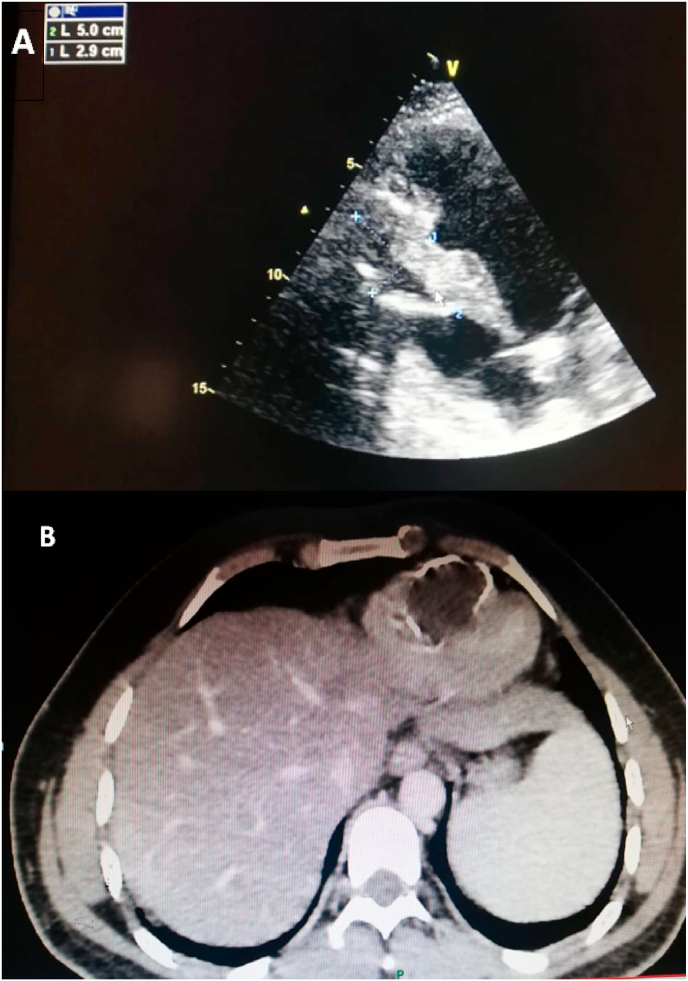
(A; B) Transthoracic echocardiography and Chest computed tomography imaging showing a right ventricular hydatid cyst.

**Fig. 2:**
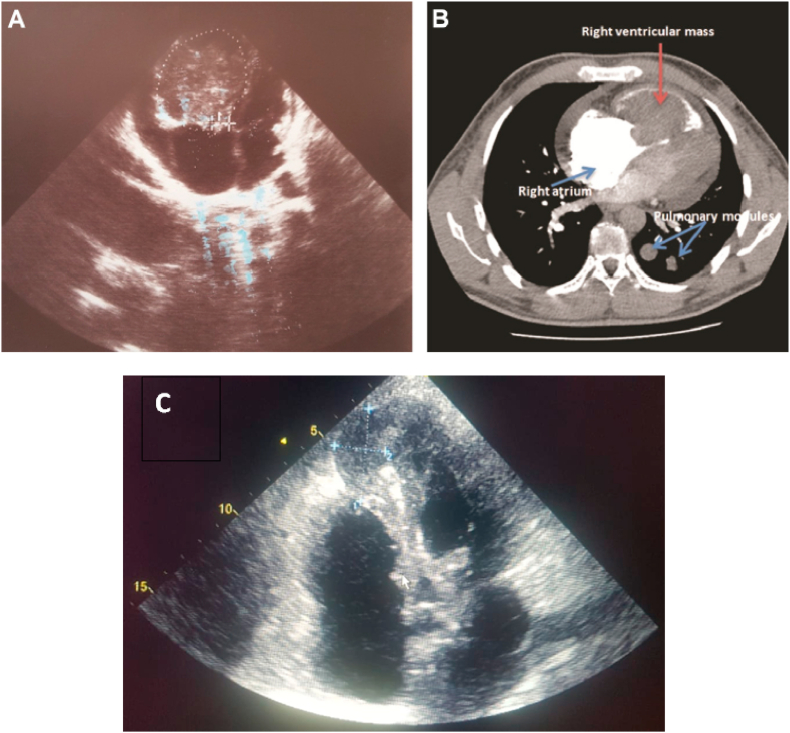
(A; B;C) Transthoracic echocardiography and Chest computed tomography imaging showing a right ventricular metastasis of a testicular non seminomatous germ cell tumor. (C) Transthoracic echocardiography imaging showing a right ventricular metastasis of a tumor infiltrated hilar and mediastinal lymph nodes.

**Fig. 3:**
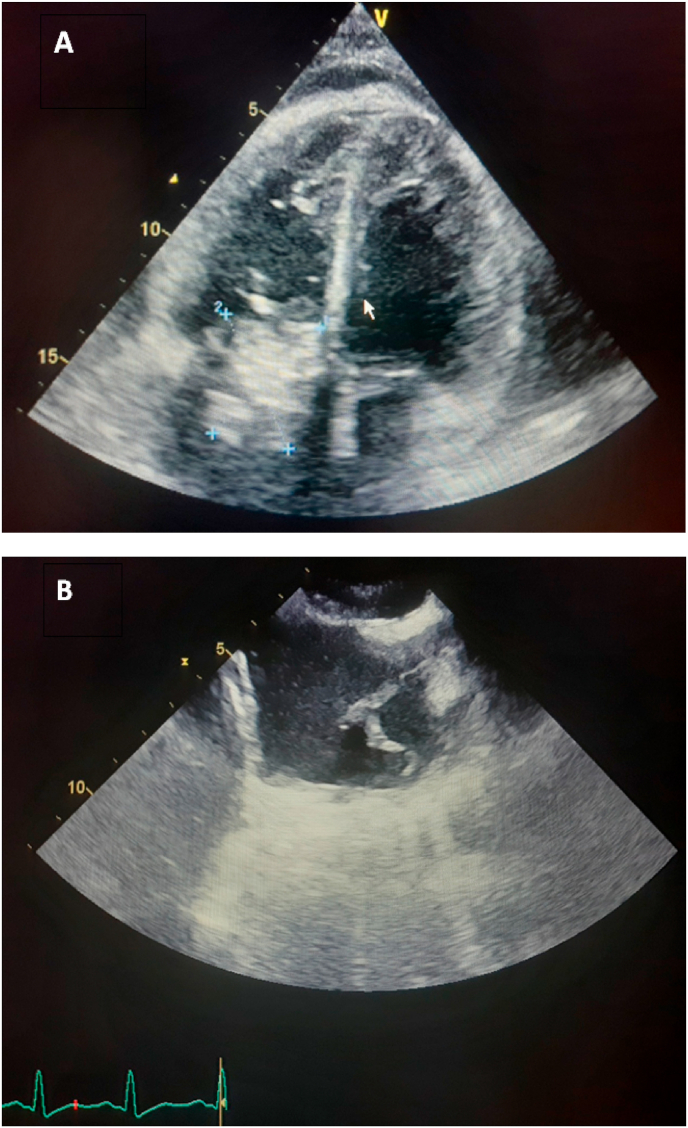
Cases of right-sidded infective endocarditis (A). transthoracic apical 4 chamber view showing vegetation attached to the tricuspid valve. (B) Transesophageal echocardiography image showing vegetation located on the lead of a port catheter.

**Fig. 4:**
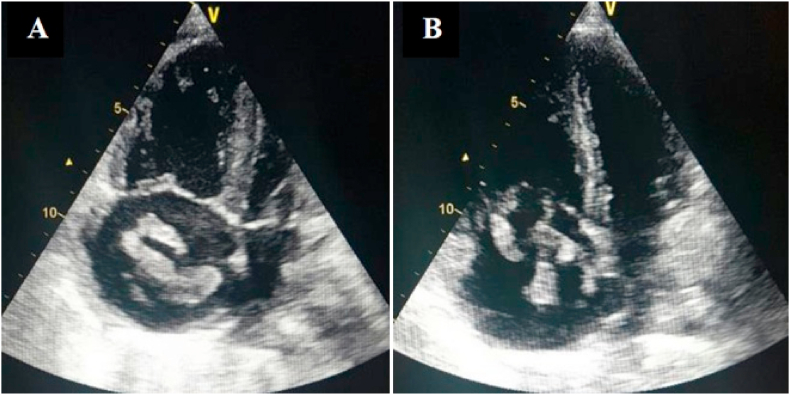
Thransthoracic echocardiography apical view showing a mobile thrombus inside the right atrium (A), protruding to the right ventriculaire in diastole (B).

**Fig. 5:**
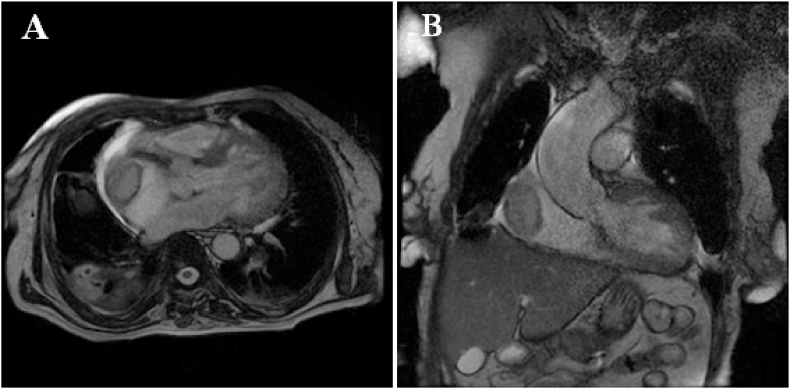
Cardiac magnetic resonance imaging showing a huge thrombus in the right atrium.

**Fig. 6:**
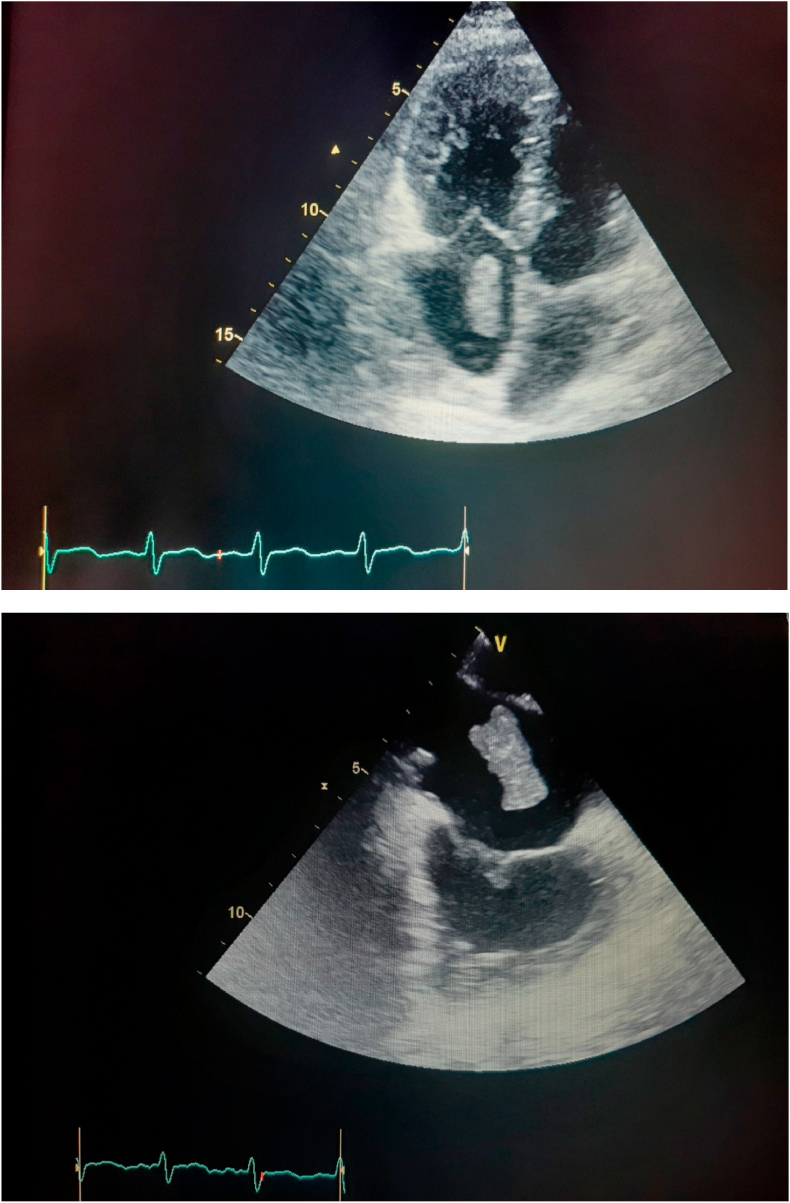
Thransthoracic (A) and transesophageal (B) echocardiography images showing a highly mobile right atrial thrombus.

The nineteen masses in the right heart consisted of eight vegetations (42.1%), seven thrombi (36.8%), one myxoma (5.3%), one hydatid cyst (5.3%) [Fig. 1 A–B], and two right ventricular metastatic lesions, one from a tumor infiltrated hilar and mediastinal lymph nodes and another from a testicular nonseminomatous germ cell tumor (10.5%) [Fig. 2 A–B–C]. The vegetations were attached to the tricuspid valve, except in one case, the vegetation was strictly localized on the lead of a port catheter [Fig. 3 A–B], while the myxoma was situated in the right atrium. The echocardiograpic diagnosis of right heart thrombi was predicated on their shape and mobility. We identified two types of thrombi, type 1: long and thin resembling a worm or a snake, extremely mobile, free floating in the right atrium without any obvious point of attachment and sometimes prolapsed through the tricuspid valve into the right ventricle. This thrombus type was seen in four cases. Some examples are shown in [Fig. 4 A–B] and [Fig. 6]. Type 2: nonmobile, adherent to the right atrial wall, seen in two cases. The diagnosis of this entity was confirmed by CMR [Fig. 4] and histology. Four of the thrombi were in males, while three were in females. The two major etiologies of right heart thrombi were embolic in 3 cases (due to propagation of a deep vein thrombosis) and in situ in 3 cases (due to atrial fibrillation) (see Fig. 5).

42.1% (n = 8) patients with right-sided masses had evidence of pulmonary embolism, including 3 cases of septic pulmonary embolism complicating right sided endocarditis. In terms of treatment, 3 patients underwent surgery (resection of a right ventricular metastasis, complicated tricuspid valve endocarditis and resection of a right atrial thrombus). Decision to operate was also made in five other cases, but death occurred before surgery in 3 cases, and two patients refused to undergo surgery. Therapeutic abstention was indicated in one patient diagnosed with right ventricular hydatid cyst since this patient was asymptomatic and the lesion was stable and inactive. The remaining patients (n = 9) received only medical treatment given to the absence of indications for surgical intervention. Five patients with right heart thrombi were managed with anticoagulant treatment (initially low molecular weight heparin, then switched to vitamin K antagonists), one patient was eligible for intravenous thrombolysis because of the association with a massive pulmonary embolism, and one patient underwent surgery. In terms of outcome, among the seven patients, 4 patients had no right-sided thrombi on echocardiography follow up, one patient died, and one patient was lost to follow up.

Of the 19 cases with right-sided cardiac masses, five patients (26.3%) died during follow-up. The cause of death was: severe ventricular arrhythmia 5 months after a resection of right ventricular metastasis, sepsis one week after a surgery of tricuspid infective endocarditis, hemorrhagic stroke 11 days after anticoagulation therapy in patients with right atrial thrombus, and multisystem failure in two cases (right atrial thrombus and right atrial myxoma.

## 4 Discussion

The cases reported show divers aspects of right intra-cardiac masses. They highlight clinical presentation and aetiology, as well as the challenges for the diagnosis and management of these clinical entities in resource-poor settings. Cardiac masses commonly present with clinical features of obstruction, embolism, arrhythmias, or constitutional symptoms (fever, fatigue) [4,5]. In keeping with previous reports, dyspnea was the most common presenting symptom in our series (accounting for 57.9%, 11/19). The constitutional symptoms were non-specific, which can be manifested in all masses types.

In spite of the high pertinence of cardiac computed tomography (cardiac CT) and cardiac magnetic resonance (MR) imaging, echocardiography remains the first-resort for cardiac mass assessment because of its accessibility, lack of iodinated contrast material or radiation exposure, and its dynamic evaluation of cardiac masses in relation to the adjacent chambers and valves [6]. In our study, the diagnosis was confirmed by transthoracic echocardiography, which permitted the identification and the characterization of the masses, determination of their location, size, shape, attachment, mobility, as well as the presence of hemodynamic consequences. Transesophageal echocardiography (TEE) is superior to TTE, The outstanding image quality as well as the high temporal and spatial resolution allow a better visualization and identification of small tumors (<5 mm) [7]. In our series, TEE was performed in four cases. The predilection site of right cardiac masses is varied with the masse type. The present results showed that myxoma and thrombi were found mainly in the right atrium. Likewise, studies showed that right cardiac myxomas and right thrombi were also found frequently in the right atrium [8,9].

Cardiac masses include tumors (both benign and malignant), thrombi and vegetations. Thrombus is the most commonly identified intra-cardiac mass [1], it may be freely mobile, attached to the endocardial wall on a pedicle, or fully adherent to the wall and sessile. Like elsewhere, thrombi and vegetations are the most frequent right cardiac masses in our series. Pulmonary embolism may be seen up to 98% of cases of right atrial thromboembolism [10]. Many case reports, patient series, pulmonary embolism registries, and meta-analyses show a high mortality rate from massive PEs in patients with a free-floating thrombus in the right heart [11,12]. In the present study, right heart thrombi were associated with pulmonary embolism in 71.4% (5/7) including four cases of free-floating right heart thrombus. Treatment administered was only anticoagulants in 4 cases, thrombolysis in 1 case. Of these 5 patients, only one died.

Right-sided infective endocarditis (RIE) is much less frequent than left-sided infective endocarditis (LIE) accounting for 5%–10% of all cases of infective endocarditis (IE), commonly involves the tricuspid valve, It has been reported more often in patients with medical devices or intravenous drug abuse. Morbidity and mortality of RSIE are reported as less serious than left-sided endocarditis with a mortality rate ranging from 3 to 30% [13,14]. In our series, there were 7 cases of right-heart infective endocarditis involving the tricuspid valve, and one case of endocarditis complicating implantable port. Septic pulmonary embolism was noted in three cases. Most patients were treated with appropriate, culture guided, antibiotic therapy with a favorable outcome. The infected line was removed in the patient diagnosed with endocarditis complicating implantable port. Only one patient underwent surgery, but unfortunately died one week later from sepsis.

The exact incidence of cardiac metastatic tumor is unknown. There are many studies on the incidence of cardiac metastases, and most of them were derived from autopsy series. Metastases may originate from blood dissemination of cancer cells, direct extension via adjacent tissues, or propagation via the superior or the inferior vena cava to the right atrium [15]. Our findings showed a low incidence of cardiac metastases (two cases).

Cardiac myxomas are the most common primary cardiac tumor in adults, typically presenting between the ages of 30 and 60 years and more commonly in women. The most common site is the left atrium which accounts for 75% of cases, proximately 10% of cardiac myxomas occur in the right atrium [16]. In the index case, a heterogeneous mobile mass with a smooth surface goes in favor of myxoma [16]. However, a histo-pathological examination following surgical excision is the only confirmatory test for diagnosing such masses.

Finally, Cardiac hydatid cyst is a rare localization and accounts for 0.5–2% of all hydatid disease, it most commonly involves the left ventricular (55–60%). Right ventricular is involved in 15% (Fig. 6), interventricular septum 5%–9%, and the right atrium could be involved in 3%–4% of cases. It can stay asymptomatic over long periods, or be discovered after serious complications. The most menacing complication is rupture that may result in cardiac tamponade, anaphylactic shock, systemic or pulmonary embolism or arrhythmias. The treatment is surgical associated fluoromébendazole or albendazole [17,18]. In our series, the cyst was found fortuitously in the right ventricular, since this patient was asymptomatic and the lesion was stable and inactive, a therapeutic abstention has been indicated.

### 4.1 Limitations of the study

We acknowledge several limitations with the present study given the retrospective nature, the small sample size and the absence of a histological diagnosis of most of masses.

## 5 Conclusion

Right sided cardiac masses visualized by echocardiography are most commonly due to thrombi, vegetations, or myxomas; nevertheless, a large number of other masses occur in the right heart as a consequence of benign and malignant tumors. This study highlights the need of a high index of suspicion and meticulous non-invasive imaging evaluation for an Infallible diagnosis due to the uniform clinical presentation of right sided cardiac masses.

## Ethical approval

The ethical committee approval was not required give the article type (case report). but we notice that the written consent to publish the clinical data of the patients was given and is available to check by the handling editor if needed.

## Sources of funding

None.

## Provenance and peer review

Not commissioned, externally peer-reviewed.

## Sources of funding

None.

## Ethical approval

The written consent to publish the clinical data of the patients was given and is available to check if needed.

## Consent

Written informed consent was obtained from the patient for publication of this case report and accompanying images.

## Author contribution

Lahmidi Ismahane: Study concept, Data collection, Data analysis, writing the paper. Charmake Darar Assoweh: Data analysis. Hasddiya Intissar: data validation. Bentata Yassmin: data validation. El Ouafi Nouha: data validation. Ismaili Nabila: Supervision, data analysis and data validation.

## Registration of research studies

UIN: researchregistry7649.

Hyperlink to the registration: Browse the Registry - Research Registry.

## Guarantor

Lahmidi Ismahane.
